# Successful ventricular leadless pacemaker retrieval after postprocedural dislodgment into the coronary sinus

**DOI:** 10.1016/j.hrcr.2024.05.011

**Published:** 2024-05-27

**Authors:** Patrice Carroz, Loïc Dällenbach, Grégoire Girod, Etienne Pruvot

**Affiliations:** ∗ University Hospital of Lausanne (CHUV), Lausanne, Switzerland; † Sion Hospital, Sion, Switzerland

**Keywords:** Leadless pacemaker, Dislodgment, Retrieval, Coronary sinus, Pacing impedance, Pacing threshold, Current of injury

**Key Teaching Points**

• We describe the first case of a postprocedural ventricular leadless pacemaker (LPM) embolization in the coronary sinus after successful implantation with successful retrieval without any complication, using a single femoral venous access.
• It is crucial to understand the importance of good pacing parameters during implantation of a LPM to avoid any complication and embolization of the device.
• After the implantation of the Aveir device using the delivery catheter, it is important to ensure an adequate alignment and to apply significant tension between the tip of the delivery catheter and the Aveir device during tether mode to ensure easy separation.

## Introduction

Leadless pacemakers (LPM) are an available pacing option for patients with bradycardia. These pacemakers are capsule-like devices that are completely intracardiac, positioned in the right ventricle, and typically placed at the interventricular septum. This technique has emerged as an alternative to transvenous pacemakers to eliminate the complications associated with leads and subcutaneous pockets. Until recently, LPM usage was restricted by its indications area (single-chamber ventricular only) but a new dual-chamber LPM has emerged recently to allow atrioventricular synchronous pacing and is FDA approved since September 2023.^1^ At the moment, 2 different types of LPM are on the market: the Micra (Medtronic, Minneapolis, MN), with US market release in 2016 with a passive fixation mechanism; and the Aveir (Abbott, Abbott Park, IL), with US market release in April 2022. The Aveir device differs from the Micra, being slightly longer (38 mm vs 18 mm for the Micra) and heavier (2.4 g vs 1.75 g for the Micra) and having an active screw-based fixation mechanism. In a real-world study, the Micra VVI LPM was associated with a 38% lower adjusted rate of reinterventions and a 31% lower adjusted rate of chronic complications compared with transvenous VVI pacing without any difference in adjusted all-cause mortality.^2^ Long-term results with the Aveir LPM are still lacking, as it is a new technology. Although rare, device dislodgment can happen and has been described to occur mainly between day 1 and day 14 after implant, with a dislodgment rate of 1.1% in the initial experience with the Micra. The usual embolization sites described so far are mainly the pulmonary artery or right ventricle, with no differences in dislodgment rate between devices positioned in the right ventricular (RV) apex and those in nonapical position.^3^ Regarding results with the Aveir LPM, the postprocedural dislodgment rate observed is 1.7% and has been described to occur with both the atrial and the ventricular device.^1^^,^^4^

## Case report

**Figure 1:**
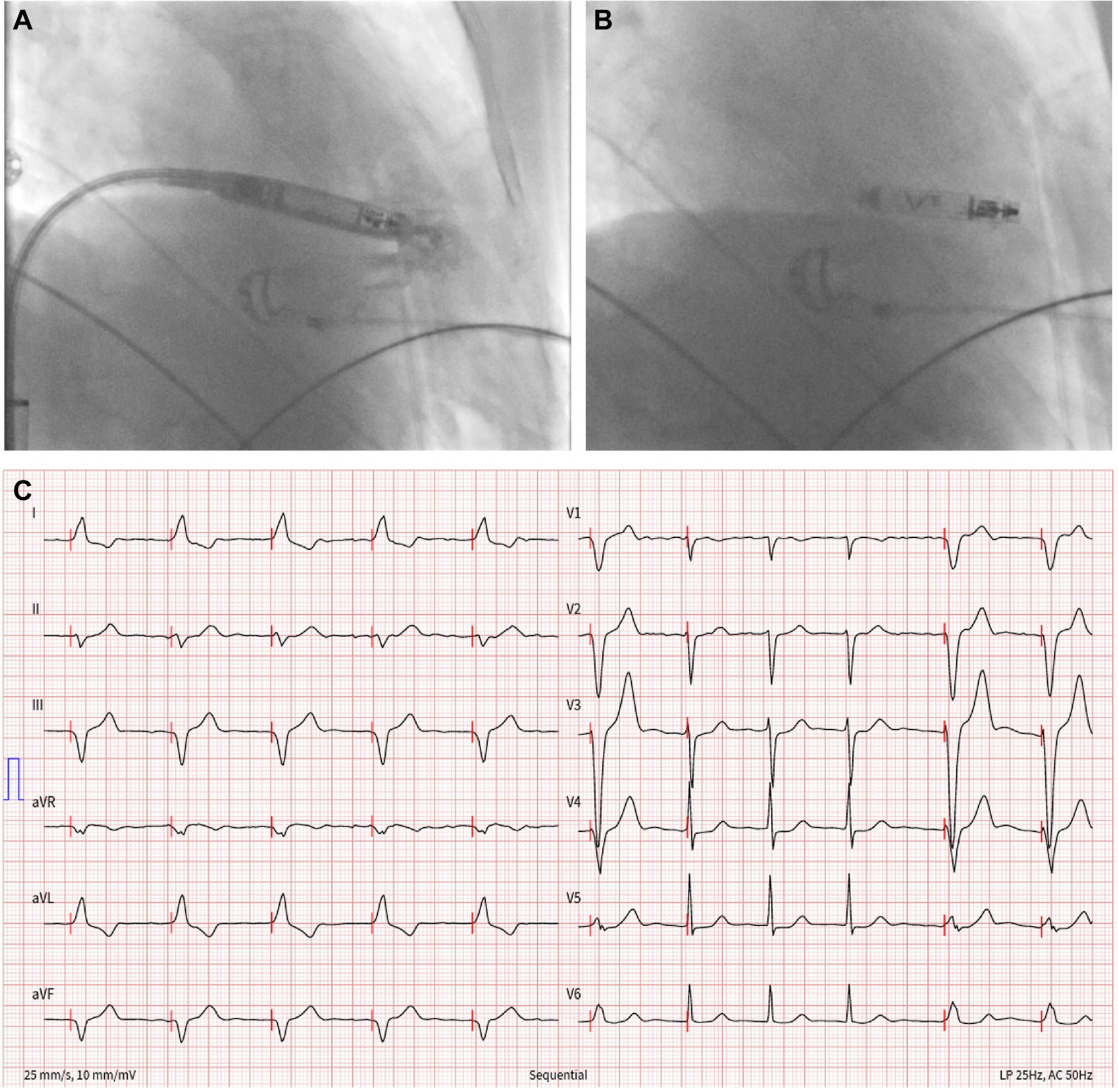
**A:** Right anterior oblique (RAO) 30° projection after contrast injection through the delivering catheter showing right ventricular (RV) septal position before delivering the device. **B:** RAO 30° projection after liberation of the Aveir (Abbott, Abbott Park, IL) leadless pacemaker showing the same position in an RV septal location. **C:** Postprocedural electrocardiogram showing good RV capture (low septal).

**Figure 2:**
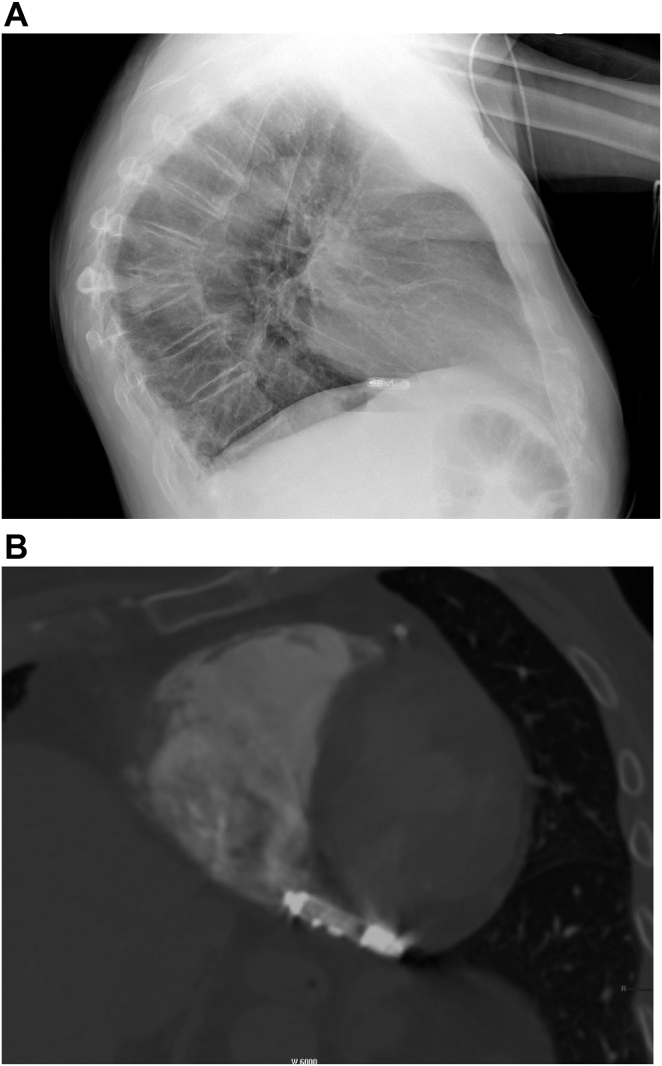
**A:** Lateral chest radiograph showing posterior dislodgment (in an uncertain position) of the Aveir (Abbott, Abbott Park, IL) leadless pacemaker (LPM). **B:** Computed tomography scan showing dislodgment of the Aveir LPM into the proximal part of the coronary sinus with the docking button protruding into the right atrium.

**Figure 3:**
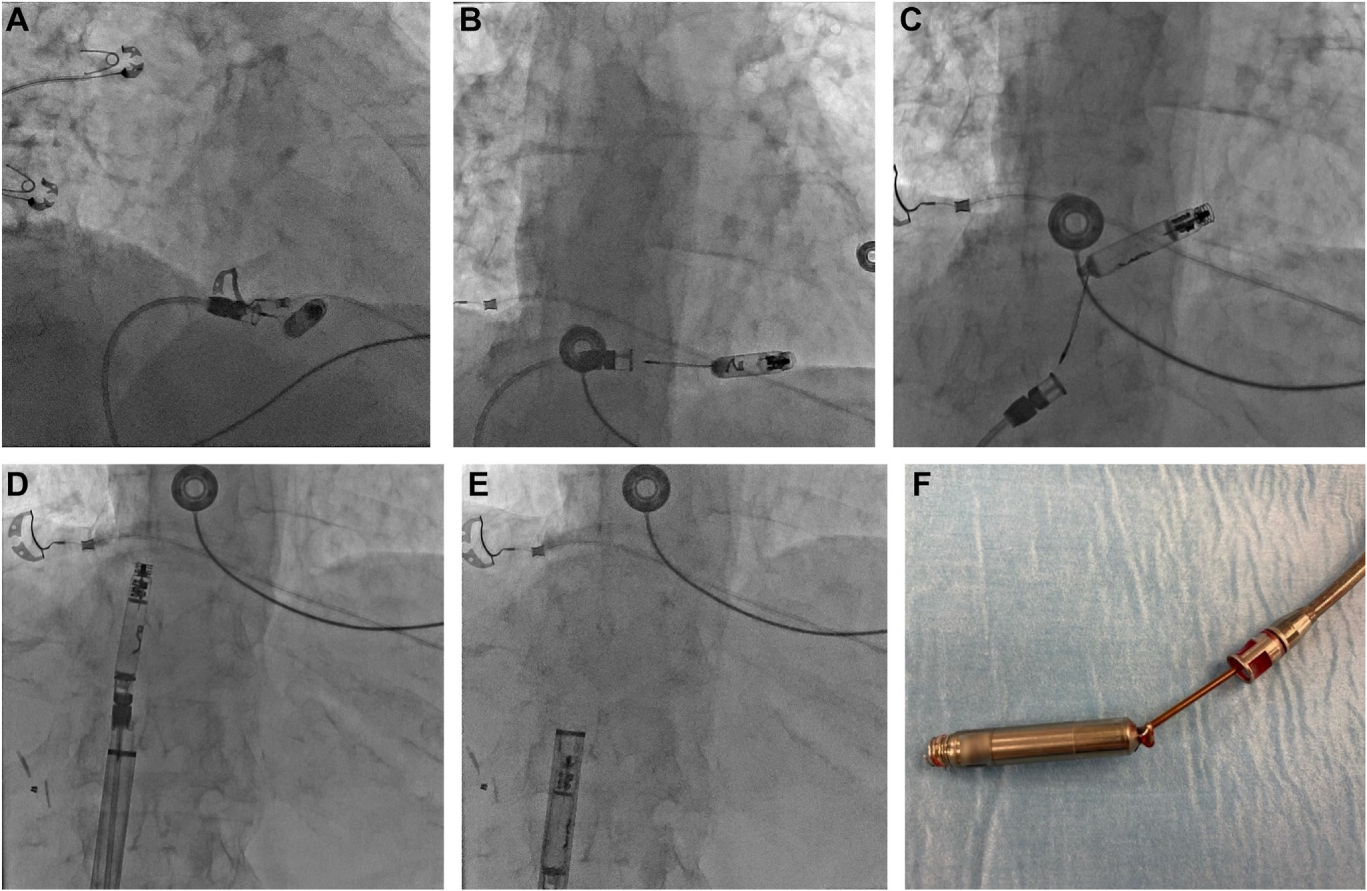
**A:** Use of the tri-loop snare retrieving catheter via a right femoral venous access, to capture the docking button (protruding into the right atrium) of the Aveir (Abbott, Abbott Park, IL) leadless pacemaker (LPM) lodged into the proximal coronary sinus. **B:** The tri-loop snare is tightened to the docking button once grabbed. **C:** The device is pulled back by gentle manual traction, without any counterclockwise rotation, into the right atrium. **D, E:** Explantation of the Aveir LPM through the 27F introducer sheath. **F:** Visual inspection of the device showing integrity of the body and of the helix.

Herein, we report the case of a 89-year old patient known for permanent atrial fibrillation who was implanted with a standard transvenous VVI pacemaker in 2003 for slow symptomatic atrial fibrillation. A generator replacement performed in November 2017 for elective replacement indicator was complicated by a pocket infection diagnosed 1 month later requiring percutaneous explantation of the system, without any complications. The patient was then not keen on having a new device implanted for the following years. On a recent Holter monitor, numerous significant symptomatic pauses reinforced the indication for a VVI pacemaker implantation. Because of the past infection and the need for ventricular pacing only, the decision was made to implant an Aveir VVI LPM. The preoperative echocardiography showed preserved left and right ventricular systolic function, no pulmonary hypertension, and light tricuspid regurgitation. This procedure was performed in February 2024 by a cardiologist experienced in LPM implantations. After venous access was obtained at the right femoral vein, an Aveir VVI LPM was successfully implanted in the interventricular septum using right anterior oblique 30° and left anterior oblique 45° projections along with contrast injections (Figure 1A and 1B), with good electrical parameters (sensed R wave 12 mV, a rise in impedance regarding the one measured before screwing the device with a value of 520 ohms, and capture threshold at 0.75 V @ 0.4 ms with a good current of injury). Some difficulty was encountered while attempting to release the device from the tethers because of some resistance. Our hypothesis is that there was not enough tension between the tip of the delivery catheter and the device body during tether mode, preventing the 2 tethers from sliding apart and thus coming out easily from the body of the LPM, preventing easy separation. The device was finally still in place once freed, with the same parameters as previously described, with stable impedance but a slight elevation in the capture threshold at 2.5 V @ 0.4 ms. After a waiting time of 10 minutes, the threshold remained slightly elevated (1.75 V @ 0.8 ms). With the other parameters in perfect range and with no radiological dislodgment, the decision was made to leave it in place. The procedure duration was 60 minutes and fluoroscopy time 20 minutes. The patient went back to the ward, and LPM check, performed 4 hours later, showed good parameters with a trend toward a decrease in the capture threshold at 1.5 V @ 0.8 ms. The electrocardiogram showed good RV capture (low septal) (Figure 1C). Twenty-four hours after implantation, ventricular capture and sensing were suddenly lost. A chest radiograph showed dislodgment of the device into an uncertain position. A computed tomography scan confirmed migration of the LPM to the proximal part of the coronary sinus (CS) in a stable position (Figure 2A and 2B). The patient remained asymptomatic, and an echocardiogram showed no pericardial effusion. The patient was then transferred to the nearest tertiary center for explantation of the dislocated device. This procedure occurred 8 days later using the dedicated Abbott percutaneous retrieving catheter system via a single right venous femoral approach. By chance, the Aveir device was lodged in the CS such that the screw was oriented toward the distal CS with the docking button protruding into the right atrium. The tri-loop snare could eventually capture the docking button after a couple of attempts. The device was then pulled back by gentle manual traction, without any counterclockwise rotation, into the right atrium. The protective sleeve was then used to cover the device before its removal through the 27F introducer sheath. Visual inspection of the device showed integrity of the body and of the helix (Figure 3A–3F). The procedure duration was 45 minutes with an x-ray time of 7 minutes and dose of 70.83 mGy. An echocardiogram performed after the procedure showed no pericardial effusion. The patient declined a new LPM implantation and was discharged home the day after the procedure.

## Conclusion

Postprocedural dislodgment of a LPM has been seldom described. Usual embolization sites included mainly the right ventricle and pulmonary artery but were never observed into the CS. We report here the first case of a postprocedural LPM dislocation into the CS with successful retrieval using the dedicated Abbott retrieving catheter via a percutaneous single venous femoral access. By chance, the Aveir LPM was positioned in a configuration that allowed easy access to the docking button with the tri-loop snare. It would have been probably more challenging if the device had to be grabbed by the distal helix. The mechanism by which the dislocated LPM ended in the CS from the RV septum remains unclear. It is important to keep in mind that good pacing parameters are crucial during LPM implantation to keep the risk of device embolization to its lowest. In our case all these parameters were good (satisfactory R wave, good current of injury, and a rise in the pacing impedance after screwing the LPM) except the threshold, which showed an acute rise following a difficult separation from the tethers. Whether this played a role in the postprocedure device dislocation in this situation remains unclear.

## Disclosures

No conflicts of interest to disclose
